# The Effect of Pimecrolimus Cream 1% Compared with Triamcinolone Acetonide Paste in Treatment of Atrophic-Erosive Oral Lichen Planus

**Published:** 2015-03

**Authors:** Atessa Pakfetrat, Zahra Delavarian, Farnaz Falaki, Mahboubeh Khorashadizadeh, Mina Saba

**Affiliations:** 1 *Oral and Maxillofacial Diseases Research Center, School of Dentistry, Mashhad University of Medical Sciences, Mashhad, Iran.*; 2 *Oral Medicine Specialist, DDS MSc, San Diego, USA.*; 3 *Department of Oral Medicine, Faculty of Dentistry, Mashhad University of Medical Sciences, Mashhad, Iran.*; 4 *Dentist, Schoolof Dentistry, Mashhad University of Medical Sciences, Mashhad, Iran.*

**Keywords:** Lichen Planus, Oral, Pimecrolimus, Triamcinolone Acetonide, Therapy

## Abstract

**Introduction:**

Oral lichen planus (OLP) is a common chronic mucocutaneous disease. Patients with atrophic and erosive types of OLP often have symptoms of soreness, and require proper treatment. The main treatment for OLP has been the administration of topical or systemic corticosteroids. The objective of this study was to compare the efficacy of adcortyl cream (triamcinolone acetonide in orabase) with topical pimecrolimus cream for the treatment of erosive OLP.

**Materials and Methods:**

Twenty-eight patients with OLP were enrolled in a single blind clinical trial and assigned to either a pimecrolimus 1% cream group or an adcortyl 0.1% cream group. The medication was applied every day for 2 months and patients were assessed every 2 weeks.

**Results:**

The mean lesion size and mean pain and burning sensation scores did not differ between the pimecrolimus and adcortyl cream groups. The pimecrolimus cream was well tolerated. No clinical drug-related adverse events were observed.

**Conclusion:**

Topical pimecrolimus cream may be recommended as a safe and effective alternative therapy in the treatment of OLP. Pimecrolimus cream is as effective as adcortyl cream in managing the signs and symptoms of OLP.

## Introduction

Oral lichen planus (OLP) is a relatively common inflammatory disease affecting approximately 0.1–4% of the adult population (1,2). OLP is found more frequently among women, and occurs most often after the fourth decade of life (3). The exact etiology of LP is still unknown, although it seems to be a T-cell-mediated autoimmune disease in which autotoxic CD8+ T-cells trigger apoptosis of the oral epithelial cells (4,5).

OLP is chronic inflammation which leads to development of a squamous cell carcinoma in 1-2% of the patients (6,7). It can be classified as either reticular, papular, plaque-like, atrophic, erosive, or bullous (4,8). Symptoms can range from none to extremely painful lesions that may interfere greatly with eating and significantly affect quality of life, especially in the atrophic, erosive, and bullous types (5,9,10). Malignant transformation is also reported to be more likely in these latter types (4,10).

Considering the role of the cellular immune system in the pathogenesis of the disease, anti-inflammatory and immune-suppressor drugs would be expected to be effective in the treatment of OLP. As there is no established cure for OLP, the first goal of treatment should be to control symptoms (10). Systemic and topical corticosteroids have to date been the most effective approach for the control of symptoms and signs of the disease, but side effects have limited their use. Indeed, some patients are refractory to this treatment and cannot tolerate these side effects (9,11).

Therefore, there is a need for an effective treatment with less morbidity and fewer side effects. The effectiveness of calcineurin inhibitors in the treatment of OLP has been suggested in some case reports and clinical trials (5,8,9,11). The calcineurin inhibitors, tacrolimus and pimecrolimus,are macrolide immunosuppressants produced by *Streptomyces tsukubaensis* and are used to prevent transplant rejection and to treat atopic dermatitis, psoriasis, and Behçet’s disease (8,12,13). Both drugs inhibit T-cell activation by inhibiting the synthesis and release of cytokines from the T-cells. They also prevent the release of inflammatory cytokines and mediators from mast cells and thereby prevent the cascade of immune and inflammatory signals (8,14).

On the basis of these characteristics and the limited number of studies investigating the effect of pimecrolimus in OLP, we decided to compare the effect of topical pimecrolimus with adcortyl on erosive-atrophic OLP in a randomized clinical trial.

## Materials and Methods

Twenty-eight adult patients with atrophic-erosive OLP as confirmed by biopsy in the accessible oral mucosa, with a lesion size less than 2 cm, and who presented or were referred to the Department of Oral Medicine, Mashhad Dental Faculty between 2008 and 2010 participated in this clinical trial. The sample size of the study population was calculated on the basis of a previously published open-label trial (8).

Inclusion criteria were biopsy-confirmed OLP in combination with a compatible clinical appearance. Atrophic-erosive lesions were limited to two sites of the oral cavity. Also, patients had received no current treatment with immunomodulatory agents. A one-week washout period was required prior to enrollment if patients were taking immunomodulatory agents.

Exclusion criteria were an inability to undergo oral biopsy for diagnosis, age younger than 18 years (13), systemic diseases or malignancy, pregnancy, lesion/lesions with dysplasia, history of allergic reaction to corticosteroids or immunomodulatory drugs. Patients with lesions adjacent to an amalgam filling were also excluded from this study.

The protocol was approved by the Institutional Ethnics Committee (IEC) of Mashhad University, and each subject signed a detailed informed consent form. Patients were evaluated by an oral medicine specialist who recorded patient demographics, medical history, symptoms, duration of disease, type, site, lesion size (cm2), and history of cutaneous lesions, among other parameters.

Patients were then randomly divided into two groups, based on a random numbering table. The control group received adcortyl (triamcinolone acetonide 0.1% in orabase, Bristol-Myers Squibb, Anagni, Italy) and the case group received pimecrolimus 1% cream (Novartis Pharmaceuticals UK Ltd). Both groups applied the cream three times a day for a 2-month period. 

After application, patients were requested not to consume food or drink for 20 minutes. Subsequently, patients were asked to rinse their mouth with 30 drops of nystatin (100,000 units) for 5 minutes for the prevention of candidiasis. 

Patient pain experience was measured using a visual analogue scale (VAS) consisting of a 10-cm horizontal line between extremes from no pain (zero) to unbearable pain (10) (Table. 1).

**Table 1:** Severity of pain according to visual analogue scale (VAS)

**Severity**	**VAS**
Very severe	7.5-10
Severe	5-7.5
Moderate	2.5-5
Mild	0-2.5

Reduction in sign scores was assessed using the Thongprasom sign scoring (Table. 2) (15).

**Table 2:** Thongprasom sign scoring for OLP

**Score**	**Characteristics**
5	white striae with erosive area≥1 cm2
4	white striae with erosive area<1 cm2
3	white striae with atrophic area≥1 cm2
2	white striae with atrophic area<1 cm2
1	mild white striae only

The size of the lesion was determined by measuring the distance between two opposite outer edges of the borders using a caliper. Two measurements, approximately 90 degrees from each other, were obtained and the largest diameter was used. Then, lesions were scored according to the scoring system summarized in Table. 2. 

Complete improvement was defined as a total absence of signs and symptoms of the lesion. Marked improvement was defined as a reduction in the size of the lesion. Aggravation was defined as an increase in the size of the lesion. Refractory was defined as no change in the size of the lesion. All patients were examined every 2 weeks for a 2-month period by a clinician who was blind to their medication and who recorded their data. Patients were aware of the medication allocated.

*Statistical Analysis*

Data were analyzed using SPSS version 11 (SPSS Inc., Chicago, IL). Inferential statistics including the Mann-Whitney test, Friedman test, Wilcoxon test and Fisher^’^s exact test were used for qualitative variables and the t-test was used for quantitative variables. In all cases P<0·05 was considered to be statistically significant.

## Results

A total of 28 patients (22 females with a mean age of 46.86±7.52 years and six males with a mean age of 42.83±17.82 years; 14 patients per group) were enrolled in this study. All patients had atrophic-erosive lesions which were limited to two sites of the oral cavity.

The two treatment groups were statistically homogenous in age, sex, cigarettes usage, drug history, presence or absence of cutaneous lesions, pain and burning sensation, location, and type of lesion on the basis of the Thongprasom criteria.

There was a reduction in the number of patients at presenting at the fourth and fifth follow-up sessions, for unknown reasons. Pimecrolimus was well tolerated with no side effects.

The mean severity score recorded for pain and burning sensation was higher in the pimecrolimus group than the adcortyl group on each visit, but according to a Mann-Whitney test, the difference was not statistically significant (Table. 3).

**Table 3:** Comparison of mean of pain and burning sensation score between the Pimecrolimus and Adcotyle groups

**Pain and burning sensation**	**Drug**	**Number**	**Min**	**Max**	**Mean**	**SD**	**P-value**
1st visit	Adcortyl	14	1	4	2.35	.84	0.069
	Pimecrolimus	14	2	4	3.71	.091	
2 nd visit	Adcortyl	14	1	2	1.71	.468	0.035
	Pimecrolimus	14	1	4	2.64	1.08	
3 d visit	Adcortyl	14	1	2	1.28	.46	0.194
	Pimecrolimus	14	1	4	2.00	1.24	
4 th visit	Adcortyl	11	1	2	1.09	.30	0.331
	Pimecrolimus	13	1	4	1.61	1.19	
5 th visit	Adcortyl	6	1	2	1.166	.40	0.914
	Pimecrolimus	4	1	2	1.25	.005	
							

A Mann-Whitney test showed no statistically significant difference in the type and severity of the lesions between the two groups, based on the Thongprasom criteria (P=0.66, P=0.91, P=0.94, P=0.30, P=0.35; respectively) (Table. 4).

**Table 4:** Comparison of lesion type in accordance with Thongprasom criteria between the two groups

**Thongprasom criteria**	**Drug**	**Number**	**Min**	**Max**	**Mean**	**SD**	**P-value**
1st visit	Adcortyl	14	2	5	2.6	1	.66
	Pimecrolimus	14	2	5	2.5	1.1	
2nd visit	Adcortyl	14	0	4	2.1	.9	.91
	Pimecrolimus	14	0	5	2.3	1.3	
3d visit	Adcortyl	14	0	4	1.6	.9	.94
	Pimecrolimus	14	0	5	1.7	1.3	
4th visit	Adcortyl	11	0	2	.9	.7	.30
	Pimecrolimus	13	0	5	1.4	1.4	
	Adcortyl	6	0	2	1	1	.35
5th visit	Pimecrolimus	4	1	2	1.5	1.5	

The mean lesion size was greater in the pimecrolimus group compared with the adcortyl group as assessed by a Mann-Whitney test, but these differences did not reach statistical significance (P=0.779) (Table. 5).

**Table 5:** Difference in mean scores between the two groups after treatment

**Clinical sign/symptoms**	**Drug**	**Number**	**Min**	**SD**	**P-value**
Lesion size	Adcortyl Pimecrolimus	14	4.35	2.00	.77
		14	4.10	2.68	
Thongprasom criteria	Adcortyl Pimecrolimus	14	1.78	.65	.53
		14	1.99	1.09	
Pain and burning sensation	Adcortyl Pimecrolimus	14	1.62	.43	.03
		14	2.26	.91	
					

Based on the Thongprasom criteria, the mean lesion-type score was higher in the pimecrolimus group, but the difference was not statistically significant (P=0.538), while mean pain and burning sensation score was significantly higher on all visits in the pimecrolimus group than the adcortyl group (P=0.031).

Both the pimecrolimus and the adcortyl groups showed a statistically significant improvement in mean pain and burning sensation score and in type and severity of the lesion score based on the Thongprasom criteria between the first and last visits. Both pain and burning sensation score and severity of lesions were reduced significantly on the fourth visit compared with the first visit in both groups. This shows that both treatments have a statistically significant effect on improving pain and burning sensation severity and signs of erosive OLP. Twelve patients in the control group (85.7%) experienced complete disease resolution while two lesions were refractory. In the case group, 10 cases (71.4%) were completely resolved, two showed marked improvement, and two cases were refractory (Table. 6).

**Table 6:** Response to treatment in both groups

**Improvement **	**Drug**	**Complete Improvement**	**Marked Improvement**	**Refractory**	**Sum**
Adcortyle group	Number	12	0	2	14
	percent	85.7	0	14.3	100
Pimecrolimus group	Number	10	2	2	14
	Percent	71.4	14.3	14.3	100
Sum	Number	22	2	4	28
	Percent	78.6	7.1	14.3	100

P=0.603

The Mann-Whitney test showed no significant differences in improvement between the two groups (P=0.603).

## Discussion

In this study, topical pimecrolimus was as effective as adcortyl in the treatment of OLP. The mean lesion size and mean pain and burning sensation score did not differ statistically between the pimecrolimus and adcortyl cream groups. The pimecrolimus cream was well tolerated with no adverse events.

OLP is a cell-mediated immunologic reaction which is driven by cytotoxic T-lymphocytes that act directly against antigens on the basal membrane (9). Different treatment options have been suggested and evaluated for OLP, including different types of topical corticosteroids, cyclosporine, tretinoin, photochemotherapy, and traditional and herbal medicines. Most of these treatment modalities have relieved symptoms but have not been shown to be effective in achieving complete remission (10,16). 

Tacrolimus and pimecrolimus act as calcineurin inhibitors, a ubiquitous calcium-dependent protein phosphatase which is responsible for immune response (8). Tacrolimus causes no atrophogenic effects compared with corticosteroids, has no effect on keratinocyte proliferation, and does not interfere with collagen synthesis (12). The long-term safety of this drug has not been proven, but blood tacrolimus levels remain within safe levels within the therapeutic range (5.0–20.0 ng/ml) (12,14). In some studies, long-term therapy with these drugs has been associated with an increased risk of malignancies such as squamous cell carcinoma and lymphoma. However, the number of these cases is small and the association has not been proven (17,18). Some patients experienced side effects such as transient burning sensation, taste alternation, and sore throat, but these were not sufficiently troublesome to cause discontinuation of treatment (9,13).

Results of this study revealed that topical treatment with pimecrolimus 1% cream elicits a similar response to triamcinolone acetonide 0.1% orabase in patients with atrophic-erosive lichen planus. No significant difference was found between pimecrolimus and adcortyl in the treatment of erosive OLP.

Different studies have been reported investigating the effect of topical pimecrolimus 1% cream on OLP, including clinical trials by McCaughey et al, Volz et al, and Passeron et al (5,9,19). In some of these studies, the rate of relapse at the location of the improved lesions was evaluated. In the Volz study, the majority of patients showed complete remission after 30 days of treatment, whereas others showed improvement after an additional 30 days (5). In studies conducted by Swift et al in 2005 and Passeron et al in 2007, a reduction in signs and symptoms were observed. However, relapse occurred after 1 month of therapy (19,20). In the present study, follow-up of patients after therapy was not performed.

In most clinical trials in which treatment with pimecrolimus was compared with routine treatment of atrophic-erosive lichen planus, pimecrolimus was well tolerated and successful in decreasing the pain and symptoms of disease. However, in some studies, no statistically significant difference between pimecrolimus and corticosteroids was observed in response to treatment (11,21). These findings are in agreement with our study. Other studies have reported side effects such as a burning sensation, ulceration and blistering at the site of ointment application (5,9,21); however, our treatment method did not produce any significant complications during 2 months of therapy.

One limitation of this study is the short duration of follow-up after remission, precluding the opportunity to assess for relapse of disease. Further studies are needed to assess treatment durability with longer duration of treatment or maintenance therapy, in order to evaluate the relapse rate.

Another limitation is the lack of measurement of pimecrolimus blood levels. In most studies, the cream was administered twice daily, whereas in the Gorouhi study it was given four times a day (5,19-21). In our study, patients were asked to use ointment three times a day.

At the present time, pimecrolimus is available in the form of an ointment, and its penetration is variable depending on the drug concentration, the vehicle, and the integrity of skin and mucous membrane. Because of the higher absorption rate of oral paste, we suggest substitution of cream with pimecrolimus orabase; a more efficient and durable treatment.

## Conclusion

Pimecrolimus cream was as effective as adcortyl cream in the treatment of OLP. Pimecrolimus can be considered a therapeutic option in cases of symptomatic OLP since it has similar therapeutic effects to corticosteroids, but probably has lower side effects and less likelihood of fungal infection. However, additional larger studies with longer treatment and follow-up periods and a more precise design in the form of placebo-controlled clinical trials are suggested. These studies should assess relapse of lesions in the improved area and probable side effects such as a burning sensation at the site of drug application.
